# Wound-healing capabilities of whale sharks (*Rhincodon typus*) and implications for conservation management

**DOI:** 10.1093/conphys/coaa120

**Published:** 2021-02-04

**Authors:** Freya Womersley, James Hancock, Cameron T Perry, David Rowat

**Affiliations:** 1 Marine Biological Association of the United Kingdom, The Laboratory, Citadel Hill, Plymouth, PL1 2PB, UK; 2 Ocean and Earth Science, National Oceanography Centre Southampton, University of Southampton, Southampton, SO17 1BJ, UK; 3 Maldives Whale Shark Research Programme, Popeshead Court Offices, Peter Lane, York, Yorkshire, Y01 8SU, UK; 4 Marine Conservation Society Seychelles, Mahé, PO Box 384, Seychelles

**Keywords:** Healing rate, whale shark, wildlife injuries, wound healing, anthropogenic activities, conservation management

## Abstract

Wound healing is important for marine taxa such as elasmobranchs, which can incur a range of natural and anthropogenic wounds throughout their life history. There is evidence that this group shows a high capacity for external wound healing. However, anthropogenic wounds may become more frequent due to increasing commercial and recreational marine activities. Whale sharks are particularly at risk of attaining injuries given their use of surface waters and wildlife tourism interest. There is limited understanding as to how whale sharks recover from injuries, and often insights are confined to singular opportunistic observations. The present study makes use of a unique and valuable photographic data source from two whale shark aggregation sites in the Indian Ocean. Successional injury-healing progression cases were reviewed to investigate the characteristics of injuries and quantify a coarse healing timeframe. Wounds were measured over time using an image standardization method. This work shows that by Day 25 major injury surface area decreased by an average of 56% and the most rapid healing case showed a surface area reduction of 50% in 4 days. All wounds reached a point of 90% surface area closure by Day 35. There were differences in healing rate based on wound type, with lacerations and abrasions taking 50 and 22 days to reach 90% healing, respectively. This study provides baseline information for wound healing in whale sharks and the methods proposed could act as a foundation for future research. Use of a detailed classification system, as presented here, may also assist in ocean scale injury comparisons between research groups and aid reliable descriptive data. Such findings can contribute to discussions regarding appropriate management in aggregation areas with an aim to reduce the likelihood of injuries, such as those resulting from vessel collisions, in these regions or during movements between coastal waters.

## Teaser

Marine species, such as the whale shark, are being subjected to unprecedented levels of human influence in their natural habitats. They may be vulnerable to obtaining injuries while spending time in surface waters. This study explores injury characteristics and timeframes of injury recovery with a view to inform management.

## Introduction

Exogenous wound healing is an essential physiological adaptation for vertebrate species (Han *et al.,* 2005; Belacortu and Paricio, 2011; Jazwinska and Sallin, 2016). For marine vertebrates, the ability to heal from external wounds is fundamental for survival and allows animals to regain motor function and minimize any prolonged impacts of injury while also managing water-borne microflora (McCallum *et al.,* 2003). Elasmobranchs (sharks and rays) are known to exhibit injury resilience and high healing capacity (Bird, 1978; Reif, 1978; Chin *et al.,* 2015; McGregor *et al.,* 2019), which has been partly explained by their unique adaptive immune responses (Marra *et al.,* 2017). However, with current expansion of ocean-based human activities, elasmobranchs now inhabit an environment where human interactions, which can result in physical wounds, are increasing (Lester *et al.,* 2020)—a circumstance that may test their natural resilience.

The incurrence of external wounds is a natural manifestation throughout the life history of elasmobranchs (Kajiura*et al.,* 2000; Chin *et al.,* 2015). Routine natural injuries tend to be minor abrasions resulting from behaviours such as courtship, copulation or aggression (Carrier *et al.,* 1994; Kajiura *et al.,* 2000; Fitzpatrick *et al.,* 2006; Domeier and Nasby-Lucas, 2007) and can occur as early as parturition, where both the female and the neonate may conclude this process with scars (Chin *et al.,* 2015). Injuries of anthropogenic origin can result from discarded marine debris (Stelfox*et al.,* 2016; Parton *et al.,* 2019), targeted and non-targeted fishing practices (Campana *et al.,* 2009; Danylchuk *et al.,* 2014; François *et al.,* 2019), research (Heupel *et al.,* 1998; Chin *et al.,* 2015) and vessel strike (Speed *et al.,* 2008; McGregor *et al.,* 2019; Lester *et al.,* 2020). Anthropogenic injuries are likely to be more complex and destructive than those obtained naturally and have the potential to induce long-term physiological adjustments, behavioural alterations and, in the worst case, death. It is important, therefore, to establish a solid baseline understanding of the characteristics and healing processes of physical wounds, from as many origins as sample size allows, so as to better identify and inform conservation opportunities where injury instance can be managed more effectively.

Systematic assessment of wound healing in free-swimming animals is a challenge as it relies on repeated observations of recovering individuals. Furthermore, in elasmobranch research there is a distinct lack of specimens for investigation as many species are negatively buoyant which may cause carcasses to sink following lethal traumatic events (Speed *et al.,* 2008; Campana *et al.,* 2016). Consequently, there are few studies that have reported on wound characteristics and change over time in detail. Fishing-related injuries (Bansemer and Bennett, 2010; Danylchuk *et al.,* 2014; François *et al.,* 2019), the impact of tagging procedures and neonatal scar healing (Chin *et al.,* 2015) have been explored, and more recently injuries that resulted from vessel collisions were monitored over time to determine healing rates (McGregor *et al.,* 2019). However, these accounts are limited to fairly small sample sizes and as a result several knowledge gaps remain in this group. Most notably, baseline knowledge related to how the type of tissue damage (e.g. superficial break in dermal surface or tear penetrating deeper sub-dermal layers) may influence healing rates is lacking.

Baseline information on wound characteristics and healing can have broad management applications, from developing appropriate animal handling procedures (Chin *et al.,* 2015) to accurately quantifying human-related threats (McGregor *et al.,* 2019). For marine mammals and reptiles, which have been the focus of a number of targeted injury impact studies (Laist *et al.,* 2001; Hazel and Gyuris 2006; Hodgson and Marsh 2007; Rommel *et al.,* 2007; Zasloff, 2011; Redfern *et al.,* 2013; Dwyer *et al.,* 2014; Martin *et al.,* 2016), information of this kind has provided a useful base to inform sustainable management of wildlife populations. In particular, information on the amount of damage individuals can sustain without suffering mortality and improved understanding of the wound characteristics, healing capacity and long-term survivorship of elasmobranchs could greatly enhance targeted conservation. This information will be especially relevant for species that spend large portions of time in surface waters for activities such as feeding and basking [e.g. whale shark (*Rhincodon typus*; e.g. Tyminski *et al.,* 2015); basking shark, (*Cetorhinus maximus*; e.g. Sims *et al.,* 2005); reef manta ray (*Mobula alfredi*; e.g. Braun *et al.,* 2014)], where animals might be vulnerable to attaining a range of natural and anthropogenic wounds.

Presented here is a systematic assessment of the healing capabilities and recovery processes of whale sharks, which aims to provide a greater understanding of the natural resilience and recovery from wounds experienced by this species. The whale shark is the largest extant species of fish (Martin, 2007) and one of the world’s most wide-ranging marine-vertebrates (Castro *et al.,* 2007; Sequeira *et al.,* 2014; Guzman *et al.,* 2018) that utilizes a diverse range of habitats including shallow coastal waters and deep open ocean (Tyminski *et al.,* 2015). This species is commonly encountered at the surface and is a good candidate to address questions related to injury resilience and healing since their broad range overlaps with a number of areas where potentially injury inducing activities also occur (Cagua *et al.*, 2014). A variety of external injuries have been observed on whale sharks (Rowat *et al.,* 2006; Speed *et al.,* 2008; Ramírez-Macías *et al.,* 2012; Araujo *et al.,* 2014; Lester *et al.,* 2020), but few studies have explicitly explored how individuals heal from these afflictions (Fitzpatrick *et al.,* 2006; Womersley *et al.,* 2016). Long-term monitoring programmes established at key coastal aggregation sites give access to extensive historical photographic and descriptive records of individual sightings and accompanying injuries. Coastal regions with high individual shark fidelity are particularly valuable as the fine scale temporal changes of wounds can be monitored in close succession. Here, a quantitative approach of documenting wound healing utilizing photographic records of whale shark injuries over time was adopted, with a focus on those which were sighted multiple instances over a short period. The main objectives of the study were to (1) develop a standardized method of measuring wound healing in free swimming whale sharks ensuring clear classification of injuries, (2) broadly explore wound characteristics and ascertain healing rates of injuries and (3) begin to investigate what factors may influence the rate of healing.

## Methods

### Data collection

Whale shark sighting records from regions surrounding the Gulf of Tadjourah, Djibouti (11°35’ N; 42°48′ E) and South Ari Atoll, Maldives (03°28’ N; 72°51′ E) were examined for injury-healing progression cases. Dedicated yearly photo-identification surveys of varying duration have been conducted by Marine Conservation Society Seychelles (MCSS) in Djibouti and the Maldives Whale Shark Research Programme (MWSRP) in the Maldives since 2003 and 2006 respectively. Detailed information about the study areas and data collection are described in Rowat *et al.* (2006) and Riley *et al.* (2010). A thorough search of descriptive encounter data was performed to locate instances where individuals with notable scarring were sighted on multiple occasions. Each highlighted injury case consisted of information on individual shark identification (ID) obtained by comparing the unique spot markings on each flank, from the 5th gill slit to the trailing edge of the pectoral fin, using I^3^S software (Van Tienhoven *et al.,* 2007). Cases also included animal size (sampled with varying accuracy depending on technique), sex (determined by the presence or absence of claspers) and descriptions of notable features such as obvious scarring. Injury and scar encounter descriptions largely followed the standardized terminology described by Speed *et al.* (2008) with wound severity, type, location on the body and source described where possible. Cases were reviewed in further detail to highlight those where clear images of the wound were available from each encounter. Upon further inspection of injury images occasional classification and terminology inconsistencies were found and further standardization was required.

**Table 1:** Table to assist in the type and severity classification of whale shark *(R. typus)* injuries from external visual assessment in-water and using encounter photographs. Detailed type descriptions, severity conditions and photographic examples (⇒) are provided of ‘abrasion’, ‘amputation’, ‘bite’, ‘blunt trauma’, ‘abnormality, ‘entanglement’, ‘laceration’ and ‘nick’ wounds.

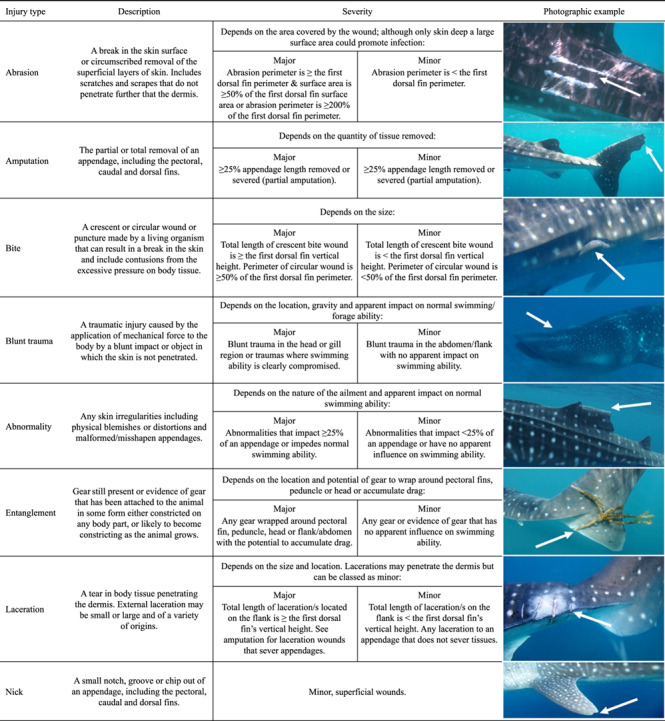

**Figure 1:**
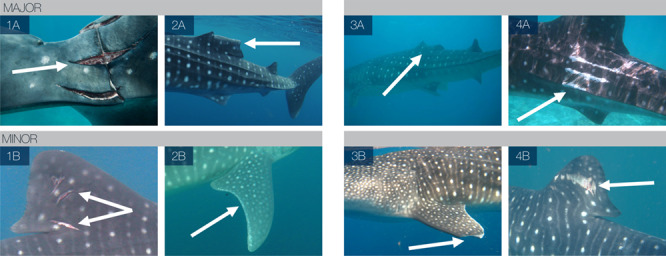
Examples of injuries (⇒) of varying type, location and severity, attributed to both natural and anthropogenic sources, portraying severity within four type groups. Images (1A)–(4A) portray ‘major’ wounds which were determined based on quantifiable characteristics (Table 1) and images (1B)–(4B) portray ‘minor’ wounds.

An updated classification system, which accounts for a larger range of injury types and a more detailed severity grouping, was developed to assist in more accurate determination of wound characteristics and improve on descriptive data in this study (Table 1, Fig. 1, and online supplementary material Table SI). In each case injury source and type were assigned by the presence or absence of key visual characteristics (Table 1). For example, clean cut incisions presenting as a parallel, repeating pattern of lacerations were indicative of a strike from a vessel propeller (Rommel *et al.,* 2007), while strikes from a vessel hull were characterized by large wounds including blunt traumas or large abrasions unlikely to result from any other natural or human cause (Laist *et al.,* 2001) (see online supplementary Table SI for examples of injuries that might result from a range of natural and anthropogenic origins). In the case of minor injuries care was taken to determine the source, although on many occasions wounds were too small to display enough notable features to accurately determine cause. Therefore, in this study minor injuries were included for comparisons of healing rate between severities but assigned as source undetermined, and source was not taken into account for the subsequent rate analysis.

 Injury severity was the final stage of assessment and based on quantifiable characteristics designed to be either accurately measured (as in this study) or estimated in water (potential future application to increase viability of in-water encounter data). Severity of each case was determined for the first injury in the photographic series. Here, severity classification was not associated with potential impact on whale shark survival, as further knowledge on the relationship between injury extent and mortality is required before any assumptions of survivorship are made. Instead, severity was related to potential impact on resource allocation and the changes in physiology and/or behaviour needed to manage the injury. Major wounds were broadly defined as ‘potentially requiring significant resource allocation or physiological/behavioural adjustments’. Minor wounds were defined as insignificant with no apparent influence on resource allocation, changes in physiology and/or behaviour.

 Key determinants for severity included a combination of descriptive indicators (wound type, location, skin colour around the wound in relation to surrounding area), quantitative indicators (relative size of the wound in relation to body features) and subjective indicators (potential influence on normal swimming and foraging ability) (Table 1 and Fig. 1). For example, in order for a laceration wound to be considered major it was present on the main body of the animal (lacerations present on appendages were considered minor, as in Fig. 1B, due to the thinner dermal layers present in these regions) and the total length of skin incisions was greater than or equal to the vertical height of the first dorsal fin (i.e. the combined length of multiple lacerations or one singular wound). If the injury did not meet these criteria but an area of discoloured skin was visible around the wound site relative to surrounding tissues or there was evidence of tissue necrosis (online supplementary material Fig. SI), the wound was considered major; this may be a sign of infection or healing complications with, as yet, unknown consequences. For abrasions to be considered major the surface area of the wound was greater than or equal to 50% of the surface area of the first dorsal fin and the perimeter was greater than or equal to that of the first dorsal fin, or the perimeter was greater than or equal to 200% of the first dorsal fin perimeter (Table 1). In Fig. 1 images (1A) and (1B) are examples of `laceration' injuries: (1A) deep laceration to the caudal peduncle region indicative of a propeller strike and (1B) superficial lacerations to the first dorsal fin of undetermined source. (2A) and (2B) are examples of ‘abnormalities’: (2A) the majority of the first dorsal fin is rolled over to the right side of undetermined source and (2B) irregular white markings on the trailing edge of the right pectoral fin of undetermined source. (3A) and (3B) portray examples of ‘amputations’: (3A) the majority of the first dorsal fin has been removed which is indicative of an anthropogenic wound (online supplementary material Table SI) that may have resulted from a vessel strike or potential opportunistic finning attempt and (3B) tip of left pectoral is missing of undetermined source. (4A) and (4B) portray examples of ‘abrasions’: (4A) abrasion covering large area of the flank indicative of a boat hull strike and (4B) superficial abrasion covering small section of the first dorsal fin of undetermined source.

These classification methods were applicable to this dataset and considered most representative of the physical characteristics of whale shark wounds likely to be assessed in water and visible in encounter photographs. Since citizen science is such a valuable input to this type of research, quantitative parameters were carefully developed to ensure they could be reasonably estimated in-water by trained personnel.

### Injury measurements

**Figure 2:**
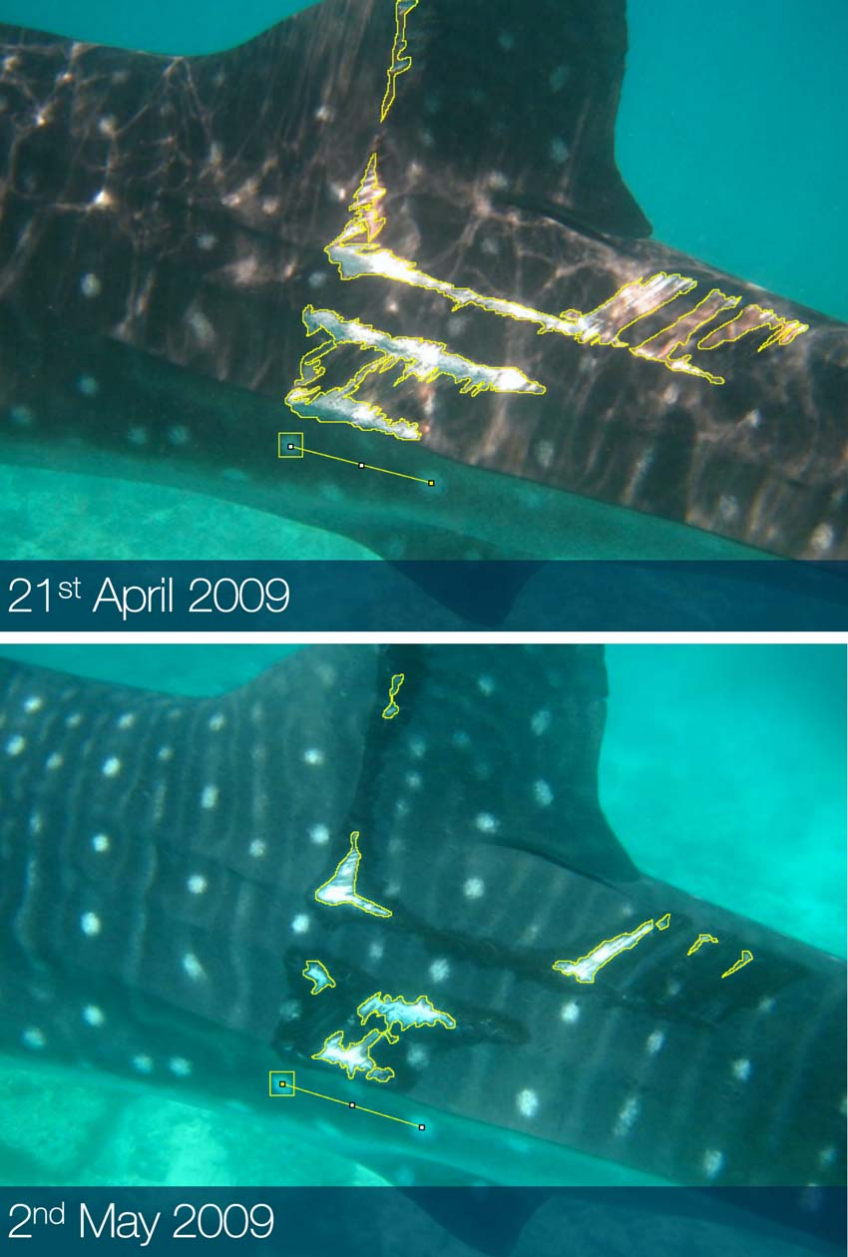
Example of methodology implemented to quantify wound change over time. The rectangle and freehand tools in FIJI by ImageJ were used to measure wound surface area and perimeter in pixels relative to the selected scale marking.

Each healing case was selected for assessment and included in the analysis if there was sufficient photographic evidence to track injury progression from an open wound to a fully healed scar, with a minimum of three images on different dates (original wound sighting, intermediate date, fully healed scar). There were three exceptions to this rule which included a case of potential appendage regeneration, which was only sighted on two instances and two minor injuries which were sighted multiple times within a 12-day period, but were not sighted with a fully healed scar. For a given image to be included in an injury progression series and used for analysis, it needed to be as close as possible to the same relative angle as other images in the series. An open wound was defined as a wound where any tissue other than the epidermis, or outermost skin layer, was visible. Thus, if any white hypodermal tissues or deeper red musculature tissues were evident in images then the injury was classed as open. A fully healed scar was defined as an injury where the epidermis had reformed, concealing previously exposed sub-dermal or muscular tissue layers. Open wounds may include regions of healing and without direct observation of the event which resulted in an injury the exact start date of wound healing cannot be accurately determined. In the absence of this data healing progression was reviewed from the first instance that an open wound was sighted rather than when the injury was obtained. As a consequence, the healing rates presented in this study represent an estimate. Injury photographs were imported into the open-source image processing software FIJI by ImageJ version 2.0.0-rc-59/1.51 k (Schindelin *et al.*, 2012). Prior to measuring, all images from each injury case were manually inspected to locate one unchanging epidermal spot marking to act as a relative scale. The rectangle tool was used to measure the surface area and perimeter of the chosen anchor point in pixels (Fig. 2).

The outline of the wound was traced using the freehand selection tool and measurements of the surface area and perimeter were taken in pixels (Fig. 2). When the wound was broken into sections, separate measurements of each section were taken and summed together to give totals. This method was chosen to provide a healing rate based on trauma that resulted from a single event and to account for wounds that began as one whole and split into smaller sections during the healing process. The total wound surface area was then divided by the surface area of the anchor point in each image to give a ratio; this allowed for standardized comparisons between image specific pixel density in each case. The same method was used for the perimeter measurements. These two-dimensional measurements did not account for total wound volume which was not possible with the dataset available. This was overcome by comparing all measurements to the first wound sighting to define relative change over time irrespective of initial injury actual size or volume. The first instance that an injury was photographed was assigned day zero and each subsequent image assigned a number of days since initial sighting. Injury measurements were compiled with associated injury locations, types, severities and individual shark ID. Healing rate was calculated on each sighting event to determine the relative rate of change between sightings at a given stage of the recovery process:

$$ {y}_i=\frac{\left(100-100\ \left(\frac{a_i/{a}_{mi}}{a_0{/}_{a_{m0}}}\right)\right)-\left(100-100\ \left(\frac{a_{i-1}/{a}_{mi-1}}{a_0{/}_{a_{m0}}}\right)\right)}{t_i-{t}_{i-1}}, $$

where *y_i_* is the measured rate on the *i*^th^ day since initial sighting, *a_0_* is initial wound surface area on first sighting, *a_i_* is wound surface area after *i* days since initial sighting, *a_m_* is the surface area of the measured relative scale *i* days since initial sighting (*a_mi_*) and on initial sighting (*a_m0_*) and *t_i_* is *i* number of days since initial sighting.

### Modelled healing rates

A minimum adequate mixed effect model was run in R package ‘lme4’ (Bates *et al.*, 2014; Team RC, 2019) to explore the effect of injury type, location and severity on the logit transformed proportion of wound change since initial sighting. Shark ID and injury case were fitted as random effects. For this analysis, surface area was chosen as the primary dependent variable and records where the injury was 100% healed were removed to standardize between injuries that were opportunistically sighted close to the time of full recovery and those that were not. This resulted in 22 usable cases with 70 observations in total. Model selection was performed by stepwise removal of non-significant terms and interactions to give a minimum adequate model (Engqvist, 2005). An exponential model was fitted using a nonlinear least squares regression from the R package ‘nls2’ (Grothendieck, 2013):

$$y(x) = a (1\textrm{- }e^{\textrm{-}bx}), $$

where *y* is the proportion of healing, 𝑎 is the value of the horizontal asymptote and *b* is the exponential rate of growth. A quadratic plateau model using a nonlinear least squares regression (Grothendieck, 2013) was also fitted to calculate on which day the rate of wound change between sightings plateaued:

$$ y(x)=\left\{\begin{array}{c}a+ bx\ \left(1-\frac{x}{2{x}_{cl}}\right), \textrm{if}\ x<{x}_{cl}\\{}a+\frac{bx_{cl}}{2},\kern4.8em \textrm{otherwise}\end{array}\right., $$

where *y* is the rate of change, *a* is the rate of change at Day 0, *b* is the exponential rate of decay and *x_cl_* represents the critical value of days (*x*) where rate of change (*y*) plateaus. For the exponential and quadratic plateau models any injury sighting that was over 150 days was omitted to gain a clearer temporal resolution; this allowed for partially healed cases to remain included but records that were observed as virtually healed after the cut off time frame were removed. Negative values were also removed from the rate plateau models. Package ‘rcompanion’ (Mangiafico, 2019) was used to assess model fit using a pseudo-r^2^ (Nagelkerke) to calculate improvements of the fitted sum of squared errors from the null.

## Results

### Injury observations

Database searches resulted in 6 suitable cases from Djibouti and 21 cases from the Maldives (online supplementary material Table SIIa, b). Although databases consisted of an extensive range of wounds of varied types from numerous sources (both natural and anthropogenic in origin), many were only encountered on one occasion or did not have suitable accompanying images for healing assessment. Of the 27 cases from 18 male individuals, the average number of images per progression series was 5.15 (± 0.49 S.E., *n* = 27). The average number of days for injuries to fully heal, not including appendage fusion or regeneration cases, was 169.57 (± 36.13 S.E., *n* = 21). Major injuries, all of which were attributed to vessel collisions (online supplementary material Table SIIa, b; injuries of alternate source, such as entanglement and bite wounds, were encountered but did not have enough appropriate images for a detailed assessment of healing), took an average of 210.5 (± 50.61 S.E., *n* = 14) days to heal and minor injuries took an average of 87.71 (± 16.69 S.E., *n* = 7) days (not including the two cases which did not reach a defined healing point). By Day 25 major injury surface area had decreased by 56.45% (± 6.06 S.E., *n* = 12), and minor injury surface area had decreased by 79.95% (± 7.60 S.E., *n* = 7). The most rapid healing case recorded was that of a minor first dorsal abrasion that reduced in surface area by 50.47% in 4 days after initial sighting.

**Figure 3:**
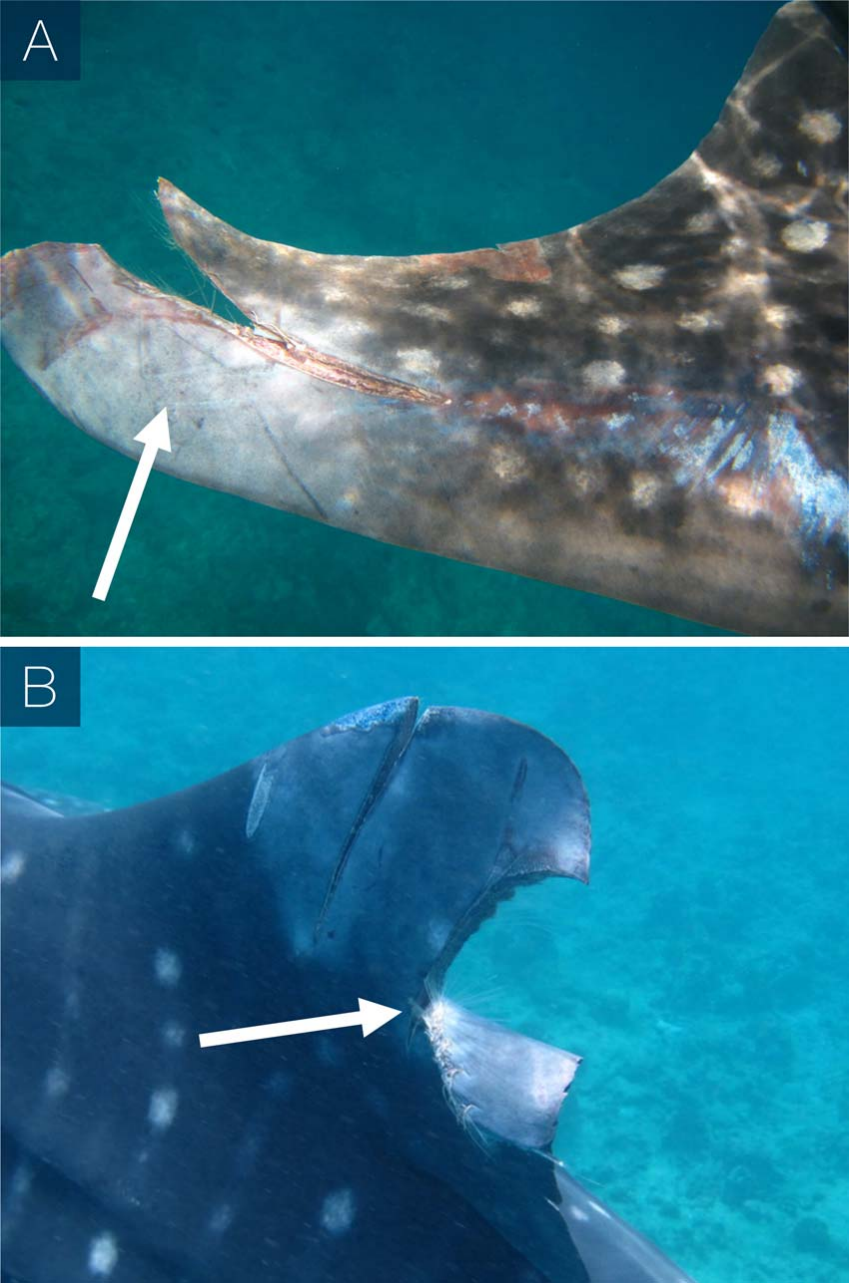
Examples of open injuries to the pectoral (A) and first dorsal (B) fins with a notable pallid epidermis surrounding the wound site and fibrous tissue present at the open wound site.

**Figure 4:**
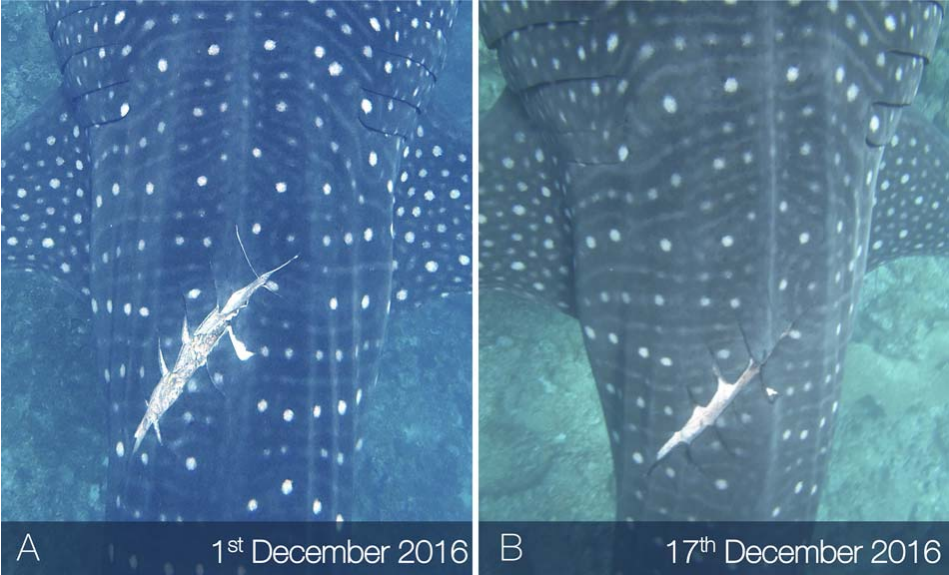
Example of the epidermis folding inward around a major laceration perimeter 16 days (B) after the wound was initially sighted (A) in December 2016.

**Figure 5:**
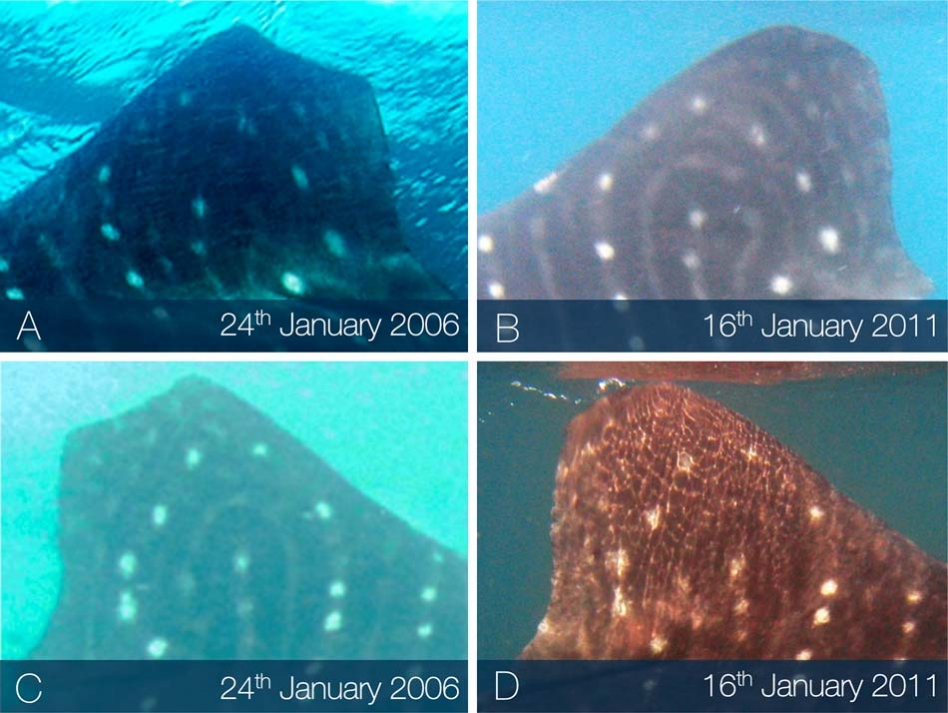
Example of appendage regeneration. In 2006 (A,C) the individual is sighted with the tip of the first dorsal fin missing. Approximately 5 years later in 2011 (B,D) tissue appears to have regenerated to fill the previously severed area and reform the natural curved shape of the first dorsal fin.

**Figure 6:**
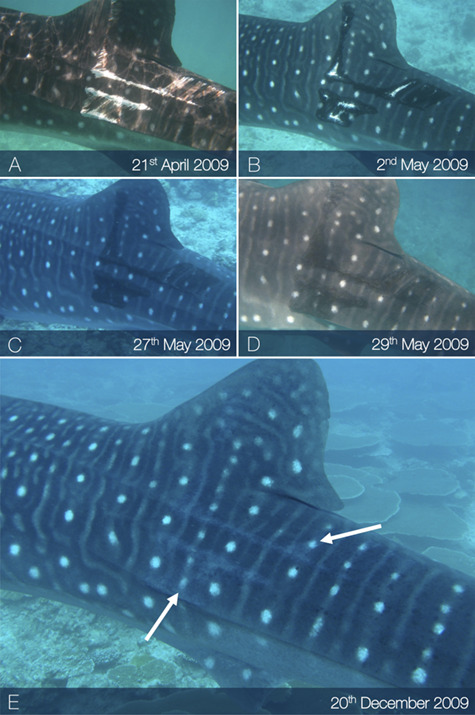
Example of white spot marking pigmentation returning to a previously wounded area over the course of ~ 5 months. Markings visible in (E) were not present on first sighting (A) where the pigmented outer skin layer had been removed.

Lacerations dissecting appendages showed signs of tissue fusion re-joining disconnected tissues (online supplementary material Fig. SII), where fibrous matter appeared to support the void left by severed appendages (Fig. 3). Lacerations to the main body showed the epidermis folding inward around the wound perimeter while the exposed sub-dermal tissue began to heal (Fig. 4). Generally, amputations showed little capacity to regrow lost tissue, however given that in many cases individuals with these afflictions were repeatedly sighted, it would seem that partial and complete fin amputations appear to have a minimal impact on short-term survivorship. In one instance a juvenile male from the Djibouti aggregation was sighted in 2006 missing the tip of the first dorsal fin. When this individual was re-sighted approximately 5 years later (2011) it appeared to have re-grown the previously amputated first dorsal fin tip (Fig. 5). The fin showed an overall surface area increase of 6.39% and a perimeter increase of 1.09% over a 3237 day (~106 month) period (Fig. 5). This is the first reported case of an elasmobranch re-growing a part of an appendage, and was not noted, to this degree, in any other case. Without a more in-depth tissue analysis it cannot be said as to what extent tissue regeneration is involved in the processes of healing in this species; however, it was observed that the tissues reforming around wounded areas, whether they be scar tissue or regenerated tissue, appeared melanized (darker) in relation to natural body pigmentation in most cases and showed some lightening over time. Black pigmentation marks often appeared in the scar region and in a few instances the white spot markings were shown to reform on a previously wounded area (Fig. 6). When seemingly recent injuries were observed with blood still visible, the outer tissue layer around the wound site appeared pallid, which may be a sign of infection (Fig. 3); however, without an improved knowledge of established signs of septicity in free-swimming elasmobranchs, such as ulceration and inflammation in captive individuals (Garner, 2013), this observation remains inconclusive.

**Figure 7:**
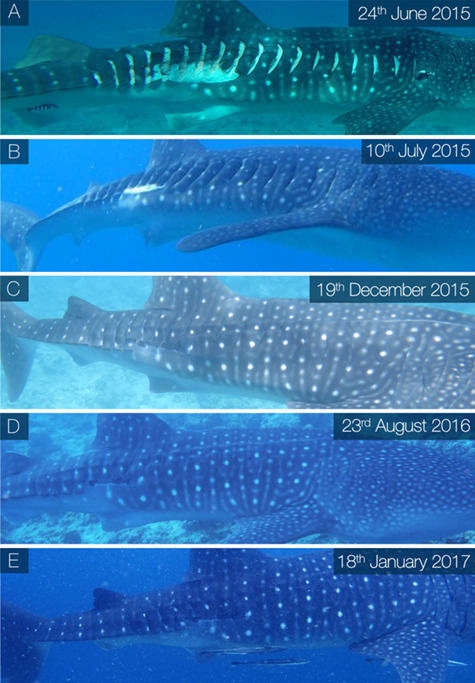
Example of an individual (WS198) which was sighted with a series of open vessel-induced lacerations to the right flank on 24 June 2015 (A). Less than 1 month later (B) the wounds showed signs of extensive healing with considerably less sub-dermal tissue visible and after approximately 6 months (C) the injury was classified as fully healed with the pigmented epidermal layers covering all tissues- In the years following (D and E) the scar showed little further change other than mild pigmentation alterations.

**Figure 8:**
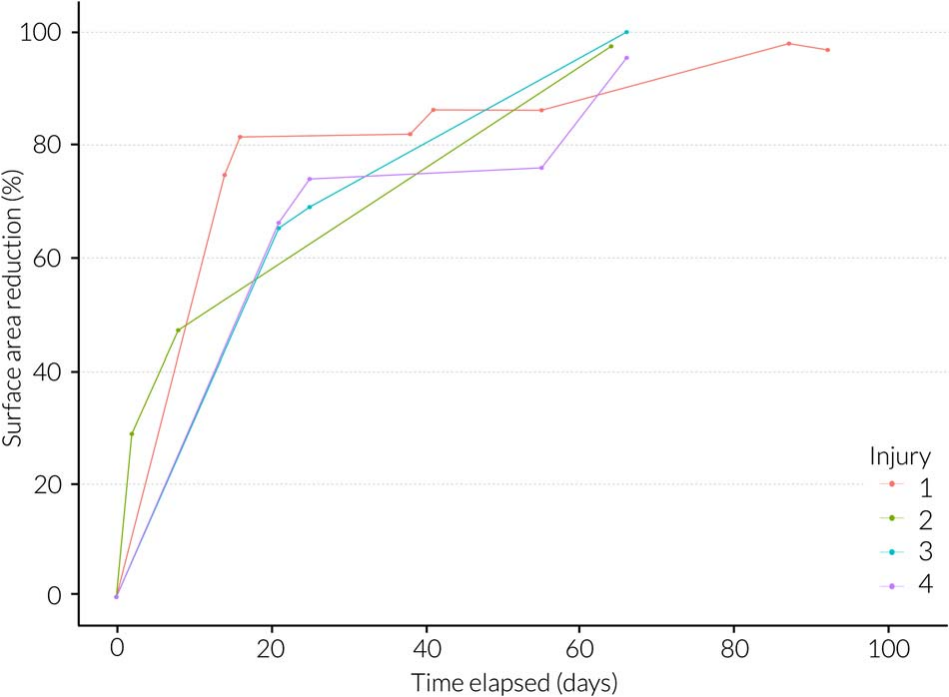
Temporal evolution (days since initial wound sighting) of wound healing showing total surface area reduction (%) of four distinct vessel-related injuries inflicted on WS198 over a three-year period.

A case study of a juvenile male whale shark (WS198, ~ 4 m in total length upon first sighting) from the Maldives dataset, which was repeatedly observed throughout the healing process, is presented here (Fig. 7). The individual was encountered on 1 June 2015 with no apparent injuries. When re-sighted 23 days later (24 June) the individual had incurred a series of major lacerations to the right flank. Owing to the large, clean cut incisions which presented as a parallel pattern of lacerations, the injury was attributed to a propeller strike (online supplementary Table SI)(Rommel *et al.,* 2007; Dwyer *et al.,* 2014), possibly by an outboard motor, which are commonly used by tourist vessels in the surrounding area (Cagua *et al.,* 2014; J. Hancock, personal communication). The lacerations were estimated to be at least a couple of days old (maximum 23 days based on pre injury encounters) as there was no blood visible and the outer epidermis had begun to fold inward around the wound perimeter. The collective wound measurements equated to 88% of the first dorsal fin surface area and 430% of the perimeter. This was the maximum wound extent relative to the first dorsal fin measured and was therefore the largest wound a whale shark was recorded surviving in this dataset. After 16 days (10 July) the wound showed signs of extensive tissue repair; most of the lacerations had fused together and little exposed sub-dermal tissue remained, however the individual appeared progressively more emaciated over the course of early observations. After a maximum of 6 months (159 days) the injury had fully closed and was classified as healed. From this date onward it showed little change, but based on visual estimates, the individual looked more robust and in improved condition. WS198 was repeatedly encountered in the years following and went on to obtain three major vessel-related injuries (one on 27 March 2016 and two on 10 May 2017) involving multiple lacerations, suggesting three distinct collision events in a three-year period. By relative Day 66 of the recovery process of the three subsequent injuries, wound surface area had reduced by 97.48%, 100% and 95.44% respectively (Fig. 8).

Cases of tissue fusion were quantified (online supplementary material Fig. SII). This included three instances of a severed first dorsal fin where appendage fusion occurred as wound margins were drawn together. In these instances, although open wounds closed, there was no defined or standardized point of healing and therefore a linear measurement of tissue reattachment was adopted to quantify the percentage reattachment of total severed length over time. Tissue reattachment occurred an average of 82.8% (± 4.7 S.E., *n* = 3) along the original severed length, with a small nick remaining in all cases. By Day 125 an average of 57.31% (± 10 S.E., *n* = 3) of tissue had reattached.

### Modelled healing rates

**Figure 9:**
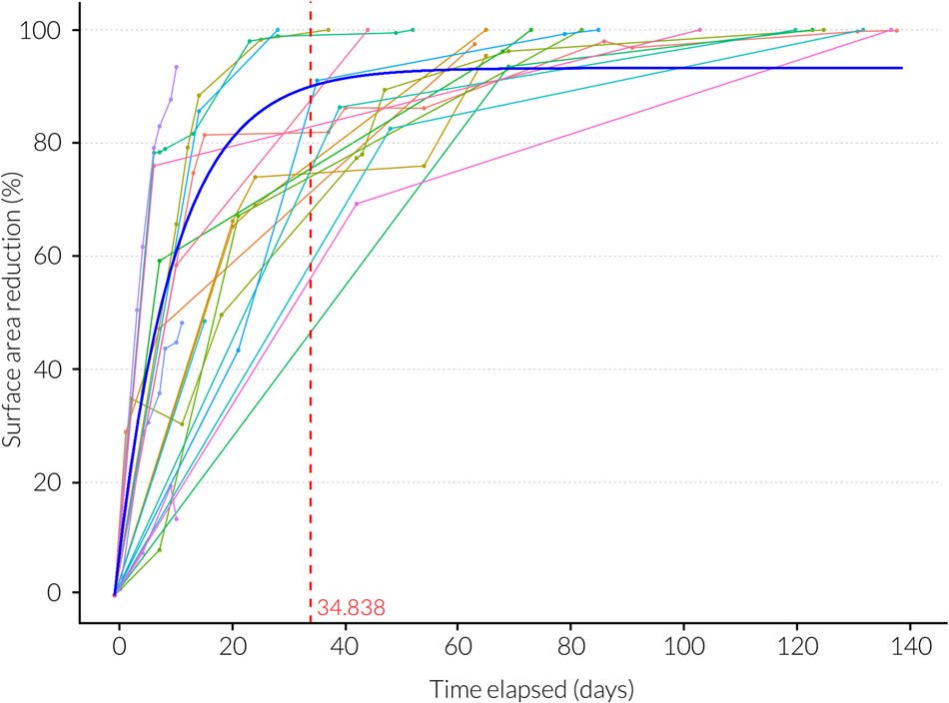
Temporal evolution (days since initial wound sighting) of wound healing showing total surface area reduction (%). Best fit exponential model is shown (blue line) where the x intercept equals 34.84 days when 90% healing is reached (red dotted line).

**Figure 10:**
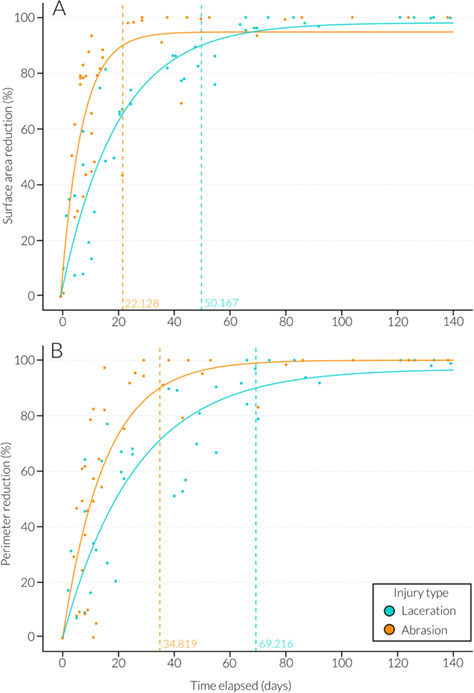
Temporal evolution (days since initial wound sighting) of wound healing showing (A) total surface area reduction (%) for lacerations (teal) and abrasions (orange) where x intercepts are 50.17 (laceration) and 22.13 (abrasion) days when 90% healing is reached (dotted line) and (B) total perimeter reduction for lacerations (teal) and abrasions (orange) where x intercepts are 69.22 (laceration) and 34.82 (abrasion) days when 90% healing is reached (dotted line).

**Figure 11:**
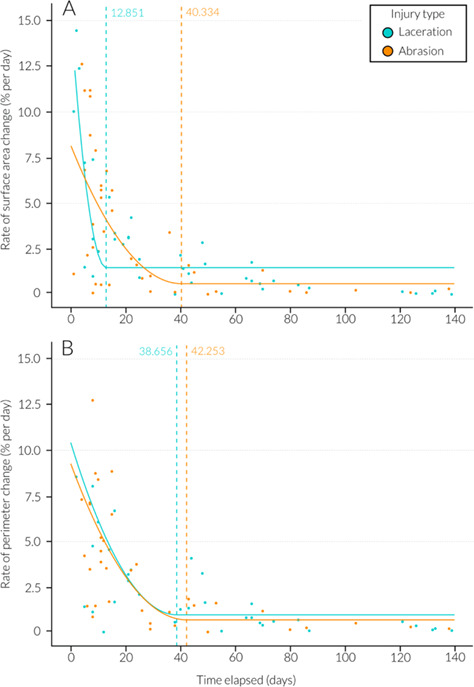
Temporal evolution (days since initial wound sighting) of wound healing showing rate of (A) surface area change between sightings (%) on a given day for lacerations (teal) and abrasions (orange) where critical x intercepts are 12.85 (laceration) (*P* < 0.001***) and 40.33 (abrasion) (*P* < 0.05*) days (dotted line) and (B) perimeter change between sightings on a given day for lacerations (teal) and abrasions (orange) where critical x intercepts are 38.66 (laceration) (*P* < 0.01**) and 42.25 (abrasion) (*P* < 0.05*) days (dotted line).

Wound severity (*χ^2^ =* 0.01*,* df = 1, *P* = 0.91) and location on the body (*χ^2^ =* 3.2*,* df = 2, *P* = 0.202) had no significant influence on the proportion of surface area reduction over time. The type of injury was significant (*χ^2^ =* 5.01*,* df = 1, *P* = 0.025*), with abrasions exhibiting a higher proportion of surface area reduction in significantly fewer days when compared to lacerations. The percentage change in wound surface area since initial sighting was well fitted by an exponential model (Fig. 9 and Table 2). From the model, 90% wound surface area closure had occurred by Day 34.84 and wound perimeter closure reached 90% by Day 52.71 (Fig. 9 and Table 2). The rate of wound surface area change between sighting occasions plateaued at 33.1 days (± 6.27 S.E., t = 5.28, *P* < 0.001***, r^2^ = 0.52) and the rate of wound perimeter change plateaued at 39.93 days (± 10.04 S.E., t = 3.98, *P* < 0.001***, r^2^ = 0.44) (Table 2). When the type of injury was explored individually, surface area reached 90% closure by Day 50.17 for lacerations and Day 22.13 for abrasions (Fig. 10). Perimeter measurements reached 90% closure by Day 69.22 for lacerations and 34.82 for abrasions (Fig. 10). Rate of surface area change between sightings plateaued at 12.85 (± 2.1 S.E., t = 6.12, *P* < 0.001***, r^2^ = 0.7) days for lacerations and 40.33 (± 15.43 S.E., t = 2.61, *P* < 0.05*, r^2^ = 0.4) days for abrasions (Fig. 11). Rate of perimeter change between sightings plateaued at 38.66 (± 13.28 S.E., t = 2.91, *P* < 0.05*, r^2^ = 0.43) days for lacerations and 42.25 (± 15.83 S.E., t = 2.67, *P* < 0.05*, r^2^ = 0.44) days for abrasions (Fig. 11 and Table 2).

**Table 2:** Outputs from the exponential and quadratic plateau models. Statistical significance is indicated by asterisks (‘.’ for *P* < 0.1, ‘*’ for *P* < 0.5, ‘**’ for *P* < 0.01, ‘***’ for *P* < 0.001).

Dataset	Measurement	*a*	*b*	*X_cl_*	*X_cl_* SE	*X_cl_* T	*X_y = 90_*	r^2^
All	Area	93.251***	0.096***	na	na	na	34.838	0.837
All	Perimeter	94.428***	0.058***	na	na	na	52.713	0.816
All	Area rate	9.306***	−0.513***	33.100***	6.265	5.283	na	0.518
All	Perimeter rate	9.769***	−0.445**	39.931***	10.037	3.978	na	0.436
Abrasion	Area	94.804***	0.135***	na	na	na	22.128	0.841
Abrasion	Perimeter	100.000***	0.066***	na	na	na	34.819	0.813
Abrasion	Area rate	8.146***	−0.374.	40.334*	15.434	2.613	na	0.404
Abrasion	Perimeter rate	9.267***	−0.405.	42.253*	15.825	2.670	na	0.442
Laceration	Area	98.124***	0.050***	na	na	na	50.167	0.925
Laceration	Perimeter	96.936***	0.038***	na	na	na	69.216	0.875
Laceration	Area rate	15.087***	−2.117***	12.851***	2.099	6.124	na	0.695
Laceration	Perimeter rate	10.421***	−0.488.	38.656**	13.282	2.910	na	0.427

## Discussion

Here a detailed report on injury healing in whale sharks is presented. The successional image measurement methods and classification system proved a viable way of comparing injury change over time. Photographic data suggest that this species has a high capacity to tolerate and recover from injuries resulting in extensive tissue damage (maximum recorded 88% and 430% of the first dorsal surface area and perimeter, respectively) with many individuals surviving wounds that were unmistakably the result of a vessel collision (*n* = 16; online supplementary Table SIIa, b). Wounds showed initial rapid healing reaching a point of 90% surface area and perimeter closure by Days 35 and 53, respectively, and slowing over time following a positive exponential curve. This suggests the majority of healing takes place in the early stages of the entire healing process which took an average of 170 days. Wounds classified as major showed a decrease in surface area of 56% in 25 days. Minor injuries were shown to reduce in surface area by as much as 80% in the first 25 days, with the fastest rate recorded showing a surface area reduction of 50% in 4 days. Lacerations, which are the most likely wound types to result from a vessel propeller collision, took significantly longer to heal than abrasions. From the models, lacerations took approximately 1 month longer than abrasions to reach 90% healed, and surface area closure between sightings reached a slower rate plateau approximately 1 month earlier than abrasions (13 days compared to 40 days), extending their recovery process. As well as providing valuable insight into wound healing processes in wild marine fauna, specifically elasmobranchs, these findings also have significant implications for conservation and the application of appropriate management to limit human-wildlife conflicts.

The rapid wound healing recorded in whale sharks supports a growing body of evidence denoting that the exogenous trauma recovery mechanisms of elasmobranchs are highly developed and able to promote healing from a number of injury types. Observed rates are similar to those recorded in captive nurse sharks (*Ginglymostoma cirratum*) and leopard sharks (*Triakis semifasciata*) where dermal denticle reformation over surgically removed areas took four months (Reif, 1978). Findings also agree with systematic observations of wild black tip reef sharks (*Carcharhinus melanopterus*) where vitellin scar surface area was shown to decrease by 94% in 24 days in neonatal individuals; a bite wound on an adult closed within 3 days and was completely healed within 40 days; and a major, deep (25 cm across and 3–5 cm deep) wound from a suspected vessel collision closed fully within 27 days (Chin *et al.,* 2015). Similarly, a male sicklefin lemon shark (*Negaprion acutidens*) was photographed with a 20 cm vertical laceration on the second dorsal fin which had healed significantly within two months and was difficult to distinguish a year later (Buray *et al.,* 2009). Pelagic stingrays (*Pteroplatytrygon violacea*) have been shown to expel circle hooks and exhibit wound recovery over a 28 day period (François *et al.,* 2019) and shark bites on reef manta rays (*M. alfredi*) were reported as completely healed within 126 to 225 days (Marshall and Bennett, 2010). Much slower healing rates have also been documented in reef manta rays where a series of vessel strike wounds had closed by 37% after 33 days and 93% by Day 295 (McGregor *et al.,* 2019). Perhaps in this case healing rates are better reflected in the tissue fusion instances presented here, where 57% reattachment occurred over total severed length by Day 125 and a small nick remained in all cases. Considerably longer healing timeframes have been recorded in wild juvenile white sharks (*Carcharodon carcharias*) where a major laceration (25 cm across) caused by a boat propeller appeared to take nine months to completely heal (Towner *et al.,* 2012) and timeframes of over six months have been recorded in grey nurse sharks (*Carcharias taurus*) following hook injuries (Bansemer and Bennett, 2010).

Inherently, the rate of elasmobranch healing likely depends on individual physiology and adaptive immunology (Luer*et al.,* 2004; Marra *et al.,* 2017). Morphological adaptations such as the thicker epidermal layers exhibited in female blue sharks (*Prionace glauca*) (Nakano and Stevens, 2008) and small-spotted catsharks (*Scyliorhinus canicula*) (Crooks *et al.,* 2013) may assist in intrinsic healing capacity in the females of these species. Additionally, neonatal individuals may have a higher rate of healing than adults associated with the importance of allocating resources and energy effectively during early life stages to optimize growth and maximize survival (Chin *et al.,* 2015). Since all sharks in this study were male, sex-related differences in healing remain unexplored in this species. Rates of recovery may also be influenced by the external environment. For example, early studies on the wound healing rate of teleost fish revealed that tissue generated more quickly across wound sites in warmer conditions (Anderson and Roberts, 1975). Reduced healing capacity in cooler waters observed in grey nurse sharks was explained as a consequence of reduced metabolic rates (Bansemer and Bennett, 2010). This may also explain why white sharks exhibited healing rates of several months when recovering from minor abrasions at Guadalupe Island where temperatures range from ∼18–20 °C (Domeier and Nasby-Lucas, 2007) and a reef manta ray occupying a region between ~ 21–24 °C exhibited slower healing rates (McGregor *et al.,* 2019). Meanwhile, black tip reef sharks and sicklefin lemon sharks in tropical waters exhibit similar healing rates to the whale shark (Buray *et al.,* 2009; Chin *et al.,* 2015). Although whale sharks have been shown to oscillate throughout a broad range of temperatures and perform deep dives into the bathypelagic zone (Tyminski *et al.,* 2015), they spend a large proportion of time in shallow surface waters and have a preference for warmer temperatures (Sequeira *et al.,* 2013). These behaviours lend support to the role of temperature in the rapid healing capacity of this species, and may also go on to explain why scarred individuals are frequently encountered in warmer surface waters (Authors, personal observation). While interspecific variation or environmental factors may influence the rate and characteristics of injury recovery, little is yet known about the relative importance of these variables for wound healing in elasmobranchs.

The instance of tissue regeneration observed in this study is a novel example of an elasmobranch regenerating a part of an amputated appendage and the first recorded form of tissue regeneration in whale sharks. Although a more in-depth analysis of tissue structure is required for a complete understanding of this case, it can be noted that this unique observation was visually distinct from conventional wound healing as tissue was regrown in response to a partial amputation as opposed to the closing up of an injury site with a scar of unknown tissue composition. Similar capabilities have been observed in whitespotted bamboo sharks (*Chiloscyllium plagiosum*) which can regenerate at least two thirds of their liver (Lu *et al.,* 2013) and many species of shark routinely regenerate and replace their teeth throughout their lives (Rasch *et al.,* 2016). Complete limb regeneration is a notable physiological adaptation in some higher vertebrate species, and serves as a mechanism to regain functional use of an appendage following amputation (Han *et al.,* 2005). In amphibians, regeneration is a local response of the cells at the amputation site and results in a perfect replacement limb regardless of the level of amputation (Han *et al.,* 2005). This is unlikely to be the case in whale sharks given that from the numerous instances of partial amputation recorded, this regeneration case was unique. Therefore, no firm conclusions regarding the species’ capacity to regrow appendages can be drawn here. Factors such as the mobility of the species and dynamism of the appendage, the amount of tissue removed and the fitness of the individual likely play a part in whether appendage regeneration will take place. Nevertheless, amputation injuries should be monitored where possible to better understand this phenomenon.

The repeated observations of injured individuals prompt further questions regarding the sublethal impacts of trauma which remain largely unexplored. Here, a maximum wound extent (WS198) in relation to first dorsal fin size is provided, which can serve as an initial estimate for the amount of damage that an individual can survive. However, changes to swimming and foraging ability, internal tissue structure alterations, or infection/inflammation resulting from injuries may significantly reduce individual fitness and post-injury survival. Previously injured individuals have been shown to exhibit less evasive behaviours and be involved in longer tourism encounters than non-injured individuals (Quiros, 2007; Haskell *et al.,* 2014), which suggests that injured whale sharks may have a reduced level of agility as a direct result of their previous injuries or are selecting warmer surface waters to speed up the healing process. Perhaps the energetic costs of seeking new foraging areas while harbouring a debilitating wound forces individuals to remain close to the foraging areas where they obtained the injury, and by default in close proximity to the initial injury source. The extent to which previous injuries influence behaviour and habitat selection or the likelihood of attaining a further injury and the healing rate of subsequent injuries requires further exploration. In addition to the immediate and prolonged behavioural/physical effects of injuries, recurring human activities around sharks has the potential to influence levels of stress. Stress-related behaviours such as changing direction or diving have been shown to be more common directly following vessel and swimmer approaches, which may carry energetic costs and inhibit recovery (Montero-Quintana *et al.,* 2018). These areas would benefit from veterinarian expertise to provide independent evaluation of the long-term potential risk of injuries and recurring human activities to shark stress, health and potential survivorship. This type of work could better inform severity classifications, sub-lethal impacts and mortality estimates which would greatly enhance population level conclusions in this field.

The current study provides the first coarse injury healing timeframe for whale sharks and can be used to infer the maximum number of days since an injury was obtained based on the healing status of a wound. For elasmobranch species in general, ecological applications of this information include evaluating wound characteristics to potentially deduce date of birth for neonatal sharks based on the healing status of their vitelline scar (Aubrey and Snelson, 2007; Rowat *et al.,* 2007; Chin *et al.,* 2015) and determining the timing of copulation (Chin *et al.,* 2013; Mourier *et al.,* 2013; Chin *et al.,* 2015) or the location of key nursery areas (McCallister *et al.,* 2013). Regrettably, these applications are difficult to apply to whale shark ecology given that encountering individuals at crucial life stages, such as adults engaged in mating activities or neonates, is extremely rare (Rowat*et al.,* 2007; Ramírez-Macías *et al.,* 2017). Additionally, significant knowledge gaps in whale shark reproductive ecology impede conclusions related to these areas. Though present lack of knowledge may limit firm ecological applications, information on injury healing timeframes may have far reaching conservation outcomes for this species, most notably in relation to managing stakeholder usage in coastal aggregation sites. Given that frequency of vessel-related injuries recorded on whale sharks is increasing in coastal regions alongside mounting recreational and wildlife tourism activities (Lester *et al.,* 2020), and a high number of individuals have been observed with external injuries (Rowat *et al.,* 2006; Ramírez-Macías *et al.,* 2012; Araujo *et al.,* 2014), it follows that further management is needed to protect individuals occupying or passing through coastal areas. Regional researchers should be encouraged to collect photographic records of external injuries, including those which have already healed, with the aim of evaluating wound characteristics and documenting the entire healing timeframe. By examining the features of injuries present on individuals the source and potential date of injury infliction can likely be ascertained. This information, along with regional domain knowledge related to stakeholder use, can have important implications for better understanding local threats to whale sharks. Furthermore, in conjunction with targeted education and awareness programmes, the improved accuracy of initial injury timing estimations can be used to support marine park management strategies (Schoeman *et al.,* 2020) and strengthen attitudes towards regulation development and sustainable compliance, as well as monitoring the success of conservation initiatives.

In summary, this study indicates that whale sharks are resilient to a range of external wounds, including those that result from vessel collisions, by showing that whale sharks exhibit rapid wound healing and long-term survival from major injuries. This species has also demonstrated the capacity to regenerate tissues in order to regrow part of an amputated appendage; a previously unobserved phenomenon. These are important findings for whale shark and wider elasmobranch conservation, especially considering the whale shark is now listed as "Endangered" by the IUCN Red List (Pierce and Norman, 2016) and up to a quarter of all shark and ray species worldwide are threatened with extinction (Dulvy *et al.,* 2014). Nevertheless, the long-term effects of trauma to the overall condition of whale sharks remain undetermined and although individuals may appear resilient to external injuries, certain traumas may have less recognizable effects such as internal damage, reduced swimming capacity resulting in higher average energetic output or behavioural changes that may significantly reduce individual fitness. Similarly, it is likely that additional energy would be assigned towards healing processes, shifting allocation away from growth (Perry *et al.,* 2018), feeding or other essential aspects of the species’ aerobic metabolic scope. The post injury emancipation observed in WS198 yields anecdotal support for this statement. As a consequence, it is essential that strategies are developed to minimize the likelihood of whale sharks attaining an injury as well as reducing further stress on newly injured individuals. Future research should aim to overcome the data limitations of the current study by conducting dedicated cicatrization studies or exploring areas related to the sub-lethal effects of injuries on overall condition or behaviour. Additionally, a large-scale assessment of whale shark space use and overlap with human activity could provide vital insight into high risk regions where this species may be vulnerable to attaining injuries and where mitigation efforts could be focused. Researchers involved in long term whale shark monitoring programmes should also be encouraged to document injuries and healing rates whenever possible. The improved understanding of whale shark resilience and response to injuries along with the impact on long-term health and survival will be an invaluable asset in refining mitigation and safeguarding the future of this endangered species.

## Author contributions

F.W. and D.R. conceived the study and F.W. designed the study. F.W., D.R., J.H. and C.P. contributed to field-based data collection. F.W. reviewed and analysed the collected data and drafted the paper. All authors contributed to subsequent drafts.

## Competing interests

The authors declare no competing interests.

## Supplementary Material

Healing_Manuscript_Supp_coaa120
