# Supplementary material for: Wound-healing capabilities of whale sharks (Rhincodon typus) and implications for conservation management
Source: Conserv Physiol. 2021 Feb 4;9(1):coaa120. doi: 10.1093/conphys/coaa120 (PMC7859907; doi:10.1093/conphys/coaa120)
Supplement: Healing_Manuscript_Supp_coaa120 [file healing_manuscript_supp_coaa120.docx]

Table SI. Table to assist in the potential source and severity classification of whale shark external injuries from visual assessment with examples of injury descriptions that might result from a given origin. Determining the severity of injury was the final step after attributing the possible origin, source, sub-source and features (such as type and location) of injury on the individual. This accounted for the varying levels of severity of injury types which depended on key determinants outlined in Table 1. On occasion, injury classification cannot be determined from descriptions alone or are case specific (CS). In these cases, photographic evidence was reviewed and the determinants in Table 1 used to assign severity. If source was not clear from photographic evidence, which may be the case for some minor (Mi) wounds, wounds were classified as undetermined. Note that entanglement is classed as both a source and type of wound and can often only be confidently determined when gear remains on the animal as the features of injury after gear has left become difficult to distinguish.


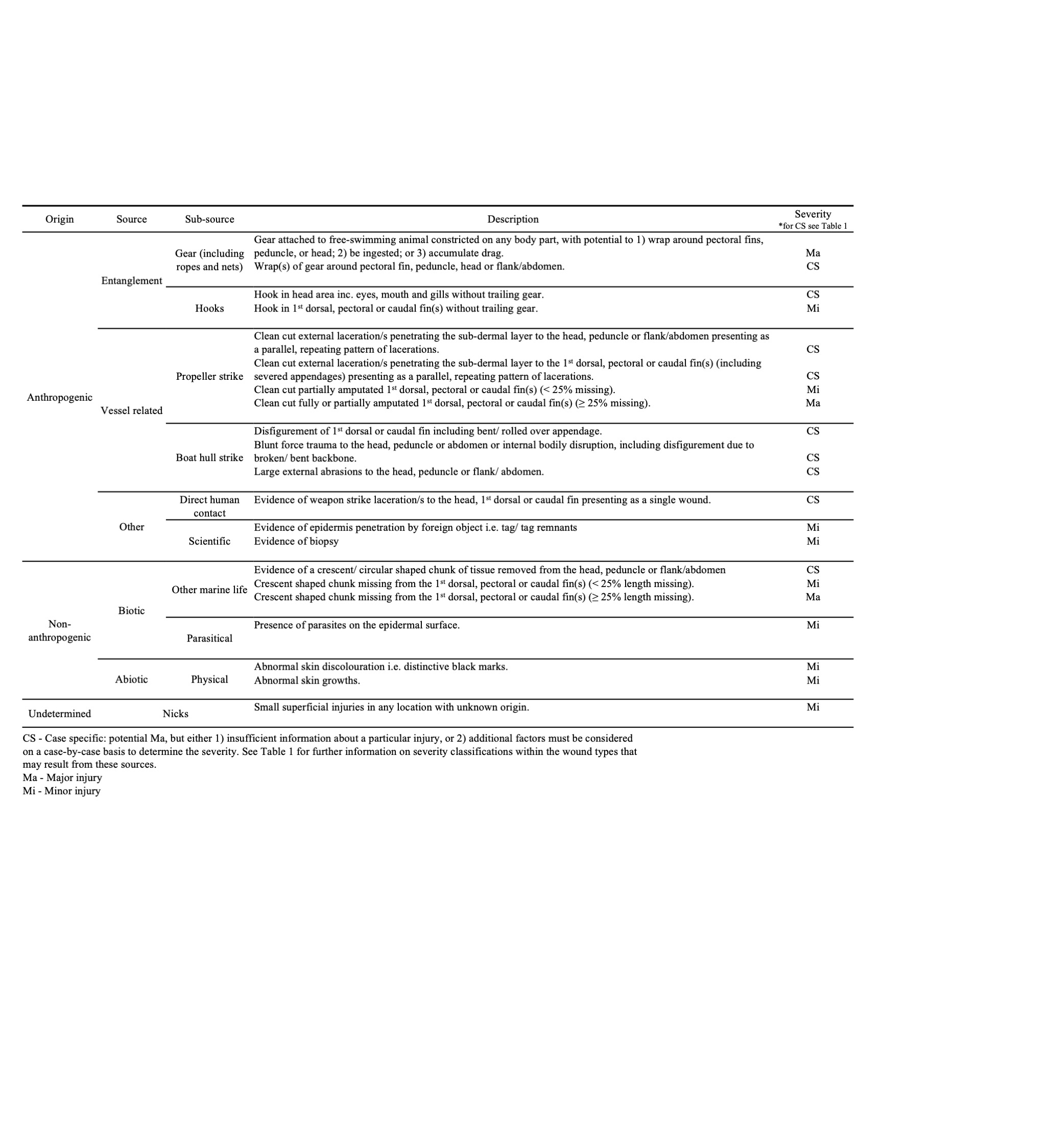


Table SII. Summaries for the subset of 27 injury cases selected to review healing from individuals encountered in Djibouti and the Maldives.

(a) Breakdown of injury characteristics.

| Injury case | Shark ID | Suitable encounters | Severity | Location | Type | Source |
| --- | --- | --- | --- | --- | --- | --- |
| 1 | WS071 | 6 | Major | Flank | Abrasion | Vessel strike |
| 2 | WS177 | 4 | Major | Flank | Laceration | Vessel strike |
| 3 | WS101 | 3 | Major | Flank | Laceration | Vessel strike |
| 4 | WS102 | 3 | Major | Flank | Laceration | Vessel strike |
| 5 | dji048 | 5 | Major | Flank | Laceration | Vessel strike |
| 6 | dji048 | 4 | Major | Flank | Laceration | Vessel strike |
| 7 | dji020 | 3 | Major | Flank | Laceration | Vessel strike |
| 8 | WS198 | 11 | Major | Flank | Laceration | Vessel strike |
| 9 | WS198 | 5 | Major | Flank | Laceration | Vessel strike |
| 10 | WS198 | 4 | Major | Flank | Laceration | Vessel strike |
| 11 | WS198 | 7 | Major | Flank | Laceration | Vessel strike |
| 12 | WS111 | 10 | Major | Flank | Laceration | Vessel strike |
| 13 | WS100 | 10 | Major | 1st dorsal | Partial amputation | Vessel strike |
| 14 | WS127 | 6 | Major | 1st dorsal | Partial amputation | Vessel strike |
| 15 | WS109 | 5 | Major | Flank | Abrasion | Vessel strike |
| 16 | WS111 | 4 | Major | Caudal | Laceration | Vessel strike |
| 17 | dji048 | 8 | Minor | 1st dorsal | Abrasion | Undetermined |
| 18 | dji048 | 7 | Minor | 1st dorsal | Abrasion | Undetermined |
| 19 | WS042 | 3 | Minor | Flank | Abrasion | Undetermined |
| 20 | WS071 | 3 | Minor | Flank | Abrasion | Undetermined |
| 21 | WS177 | 3 | Minor | Flank | Abrasion | Undetermined |
| 22 | WS337 | 5 | Minor | Caudal | Laceration | Undetermined |
| 23 | dji002 | 2 | Minor | 1st dorsal | Partial amputation | Undetermined |
| 24 | WS183 | 3 | Minor | Flank | Abrasion | Undetermined |
| 25 | WS184 | 9 | Minor | Flank | Abrasion | Undetermined |
| 26 | WS108 | 3 | Minor | 1st dorsal | Abrasion | Undetermined |
| 27 | WS220 | 3 | Minor | Flank | Laceration | Undetermined |

(b) Count and proportion of wound types in severity, location, and source classes. Values in parenthesis are proportions of all wounds within each category.

|  | | Type | | | Total |
| --- | --- | --- | --- | --- | --- |
|  |  | Laceration | Abrasion | Amputation |  |
| Severity | Major | 12 (0.86) | 2 (0.2) | 2 (0.67) | 16 (0.59) |
|  | Minor | 2 (0.14) | 8 (0.8) | 1 (0.33) | 11 (0.41) |
| Source | Vessel strike | 12 (0.86) | 2 (0.2) | 2 (0.67) | 16 (0.59) |
|  | Undetermined | 2 (0.14) | 8 (0.8) | 1 (0.33) | 11 (0.41) |
| Location | Flank | 12 (0.86) | 7 (0.7) | 0 (0) | 19 (0.7) |
|  | 1^st^ Dorsal | 0 (0) | 3 (0.3) | 3 (1) | 6 (0.22) |
|  | Caudal | 2 (14) | 0 (0) | 0 (0) | 2 (0.07) |
| Total | | 14 (0.52) | 10 (0.37) | 3 (0.11) |  |

Figure SI. Examples of potential tissue necrosis on the flank (A) and upper caudal (B) which may have healing consequences.


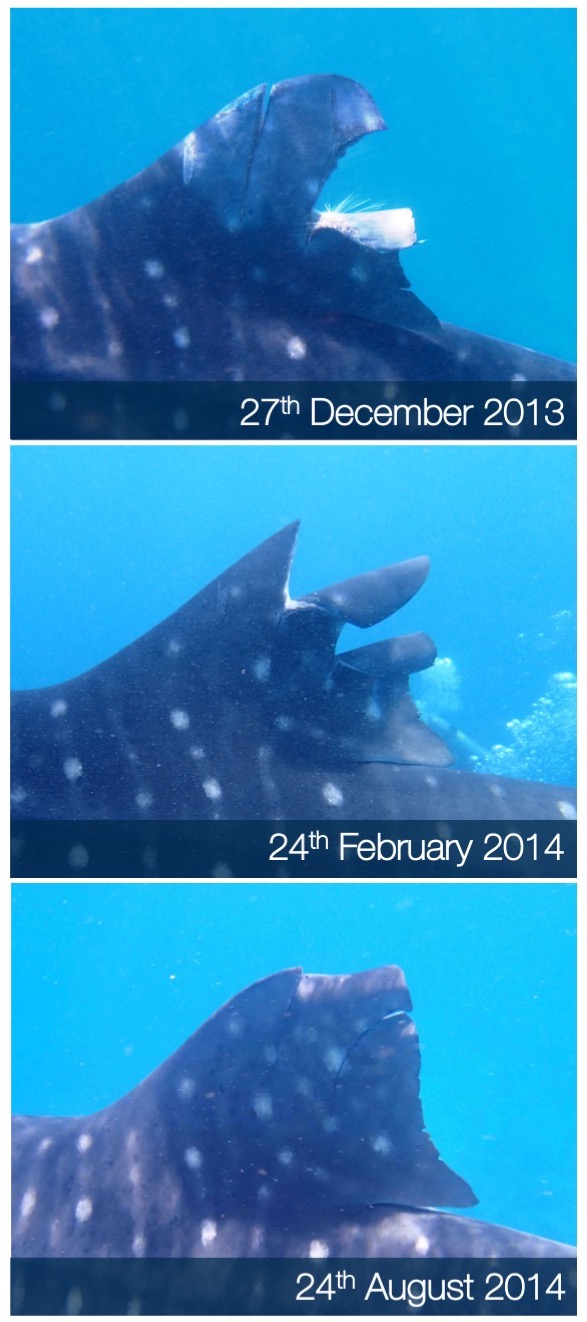


Figure SII. Example of tissue fusion where the 1^st^ dorsal fin regains natural shape and function over the course of approximately 8 months. Note pallid tissues surrounding wound site upon first sighting.
